# Brain Damage after ECT? No significant changes of Neurofilament Light Chain and Glial Fibrillary Acidic Protein in Cerebrospinal Fluid

**DOI:** 10.1192/j.eurpsy.2025.1361

**Published:** 2025-08-26

**Authors:** S. Riessland, M. Ponleitner, V. Millischer, S. Macher, R. Lanzenberger, P. Rommer, R. Frey, D. Rujescu, P. Baldinger-Melich

**Affiliations:** 1 Department of Psychiatry and Psychotherapy, Clinical Division of General Psychiatry; 2 Comprehensive Center for Clinical Neurosciences and Mental Health; 3 Department of Neurology, Medical University of Vienna, Vienna, Austria

## Abstract

**Introduction:**

Electroconvulsive therapy (ECT) is an effective treatment for major depressive disorder (MDD). Only few studies have measured CSF in patients undergoing ECT; thus far, no prognostic candidate biomarkers for treatment response have been found (Kranaster *et al.* Neuropsychobiology. 2019;77(1):13-22). Neurofilament light chain (NfL) is a protein found in the axons of neurons. If elevated in blood or cerebrospinal fluid (CSF), it is an indicator of neuroaxonal damage. A recent study including 15 patients with MDD who underwent ECT found no significant change in serum NfL concentrations after completion of treatment (Besse et al. Eur Arch Psychiatry Clin Neurosci 2024; 274(5):1187-95). The astrocyte marker glial fibrillary acidic protein (GFAP) is elevated in MDD and neuroinflammatory diseases. Serum GFAP levels in 40 MDD patients decreased after ECT compared to baseline (Xu *et al.* Psychiatry Clin Neurosci. 2023; 77(12):653-64). No studies measuring CSF levels of both markers have been performed.

**Objectives:**

In this prospective study we aimed to measure changes in CSF of NfL and GFAP in patients undergoing an ECT series.

**Methods:**

In a sample of 9 MDD patients undergoing bilateral ECT, CSF was analyzed before and after the 8^th^ ECT session. Patients took antidepressant medication in a steady state over the course of ECT. A mixed-effects linear regression analysis was done using the log-transformed NfL and GFAP levels as outcome variables. The timepoint (pre-ECT, post-ECT) were entered as fixed effects, patient ID was included as a random effect to account for individual variability. We corrected for multiple testing and defined alpha = 0.05/2 = 0.025. Statistical analyses were performed using R version R-4.3.2.

**Results:**

The mean age ± SD was 34 ± 11 years, 6 out of 9 patients (67%) were women. Mean elevations of NfL by 19,9 pg/ml (95% CI: -120.3 to 160.0) and GFAP by 445.8 pg/ml (95% CI: -1279.6 to 2171.4), there was no significant change in NfL (*p* = 0.213) or GFAP (*p* = 0.362) levels after ECT. Figure 1 shows concentrations of both NfL and GFAP pre and post ECT.

**Image:**

**Figure:**
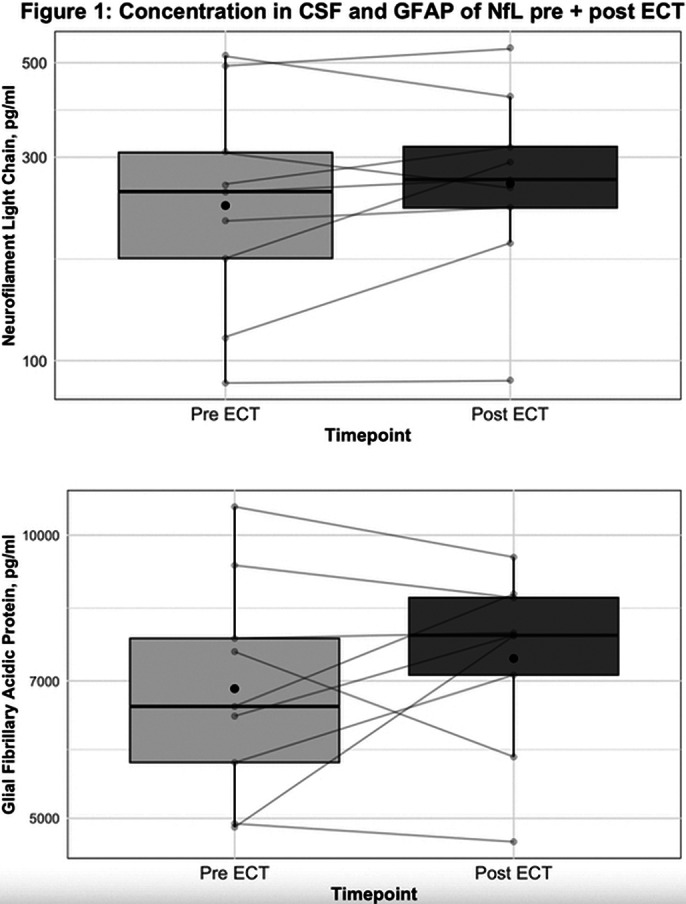

**Conclusions:**

This study found no significant changes of NfL or GFAP levels in CSF after an ECT series, suggesting no evidence of neuroaxonal damage or brain damage through astrocyte activation related to ECT. However, the small sample size may have obscured potential effects. Future research with larger sample sizes is therefore essential.

**Disclosure of Interest:**

S. Riessland Grant / Research support from: This study was sponsored by the Austrian Science Fund (project number KLI 1098, principal investigator P. Baldinger-Melich) and the 2021 NARSAD Young Investigator grant (grant number 29950, principal investigator P. Baldinger-Melich). S. Rießland is partially funded by the Austrian Science fund and the NARSAD Young Investigator Grant., M. Ponleitner: None Declared, V. Millischer: None Declared, S. Macher: None Declared, R. Lanzenberger: None Declared, P. Rommer: None Declared, R. Frey: None Declared, D. Rujescu: None Declared, P. Baldinger-Melich Grant / Research support from: This study was sponsored by the Austrian Science Fund (project number KLI 1098, principal investigator P. Baldinger-Melich) and the 2021 NARSAD Young Investigator grant (grant number 29950, principal investigator P. Baldinger-Melich). The other authors have no conflict of interest related to this study to declare.

